# Thoraco-abdominal Ectopia Cordis in Southwest Cameroon

**DOI:** 10.11604/pamj.2014.18.124.4247

**Published:** 2014-06-08

**Authors:** John B Chishugi, Trixy J Franke

**Affiliations:** 1 Mount Mary Hospital,Diocese of Buea, Buea, Cameroun; 2 Buea Seventh-day Adventist Health Centre, Buea, Cameroon

**Keywords:** Ectopia Cordis, congenital defect, Cantrell's pentalogy

## Abstract

Ectopia cordis is a rare congenital defect where the heart is completely displaced outside the chest wall. Cantrell's pentalogy is an embryologic anomaly with five classic midline deficiencies often associated with ectopia cordis. Here we present a case of thoraco-abdominal ectopia cordis, brief literature review, and possible implications for changes in antenatal care.

## Introduction

Thoraco-abdominal ectopia cordis is an uncommon congenital malformation that can present anywhere in the world. It is often associated with Cantrell's pentalogy of anomalies. Treatment usually consists of staged-surgical repair in highly specialized medical centres. The prognosis is historically poor although advances in early diagnosis and surgical technique have led to improved survival. Early diagnosis through obstetric ultrasound would allow for improved informed clinical decision making on the part of the physician and family.

## Patient and observation

A 23-year-old G3P1010 at 41 + 1 weeks presented with spontaneous rupture of membranes and regular contractions. Due to an increasing foetal heart rate and thick meconium, the patient subsequently underwent an urgent caesarean section for foetal distress.

The mother's gynaecological history was significant for a spontaneous abortion at 4 weeks and an intrauterine foetal demise (unknown cause) at 27 weeks with a subsequent uncomplicated vaginal delivery of the expired foetus. There was no family history of congenital abnormalities.

The mother had no ultrasound during the current pregnancy. She had no known exposure to teratogens. At 25 + 4 weeks she was treated for severe malaria with intramuscular Artemether. She received the normal tetanus vaccinations, sulfadoxine/pyrimethamine malaria prophylaxis, and folic acid + iron vitamins during her pregnancy. Other than mild anaemia (Hb 10.5 g/dl) she appeared to have an uncomplicated pregnancy.

The neonate at time of caesarean had a nuchal cord wrapped round his neck and head. His APGARS were 8, 10, & 10 at 1, 5, and 10 minutes, respectively. He weighed 3.5 kilograms with a head circumference of 34 centimetres and a length of 50 centimetres.

**Figure 1:**
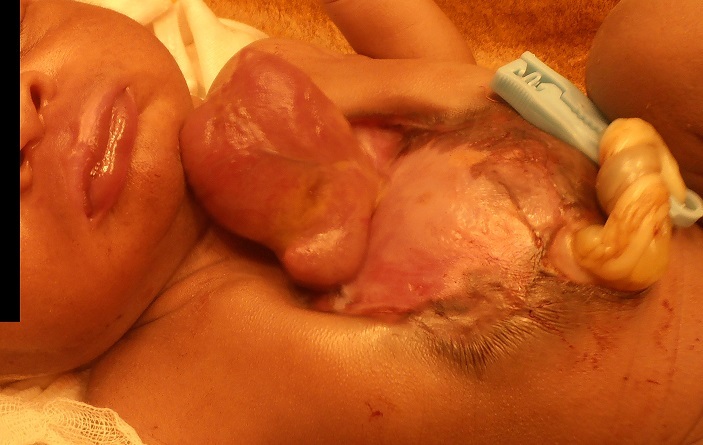
Male infant, weighing 3.5 kg, minutes after caesarean delivery with complete ectopia cordis, absence of the sternum, anterior diaphragm, and subraumbilical abdominal wall. Vigorous at birth but expired during transport to tertiary facility

He appeared vigorous with spontaneous breathing and excellent tone on examination. The sternum was absent and the heart was completely outside the chest, covered with a thin serous membrane. The upper abdominal wall and anterior diaphragm were both absent, presenting an open abdominal wall defect with a three-vessel umbilical cord that inserted into the lower abdominal wall. The chest wall rose symmetrically with respiration. No echocardiography was performed to diagnose any intracardiac defects. Findings were consistent with a thoraco-abdominal ectopia cordis. The infant was immediately referred to a tertiary paediatric hospital in the country's capitol but died en route within hours. No autopsy was performed (Figure 1).

## Discussion

Ectopia cordis is a rare congenital malformation. An incidence of ectopia cordis was reported to be 5.5 to 7.9 per 1 million live births by Chandran S. et al [1]. Alphonso N. et al cited the same incidence but limited it to thoracic ectopia cordis [2]. Likely there arises some confusion in the literature due to variations in the definition of partial and complete ectopia cordis, as noted by Firmin RK, et al., and also the infrequency of this often fatal malformation [3].

Ectopia cordis is postulated to start early in the embryonic period around the 8th and 9th week of pregnancy [4]. Kumar B, et al. propose that even as early as the 14-18th day of embryogenesis, defective formation and differentiation of the ventral mesoderm has already begun [5]. Complete or incomplete failure of midline fusion results in a range of related malformations from isolated ectopia cordis to complete ventral evisceration [4].

Cantrell JR, et al. are thought to be the first to describe a pentalogy of malformations in 1958, of which ectopia cordis is often associated. Cantrell's pentalogy consists of (1) midline supra-umbilical abdominal wall defect; (2) defect of the lower sternum; (3) deficiency of the anterior diaphragm; (4) defect in diaphragmatic pericardium; (5) congenital intracardiac defects [6]. Ectopia cordis occurs when the heart is displaced outside the chest wall [5]. The displacement of the heart can be cervical, cervico-thoracic, thoracic, throaco-abdominal, or abdominal. The most common types are thoraco-abdominal and abdominal [2].

Kumar B, et al. cite that variations of Cantrell's pentalogy can be classified according to the number of defects found: Class 1 composes an exact diagnosis where all five of the defects are present; class 2 is deemed a probable diagnosis when four, including intracardiac and abdominal wall defects, are present; class 3 is termed an incomplete diagnosis when at least two of Cantrell's anomalies are noted where one is sternal [5]. According to this classification, our case falls into the third since, although four of the defects were noted (supraumbilical, sternal, diaphragmatic, pericardial) no echocardiography or autopsy was performed to confirm an intracardiac defect.

Both intracardiac and other associated anomalies have been reported with ectopia cordis. In order of decreasing prevalence, intracardiac defects include ventricular septal defect, atrial septal defect, tetralogy of Fallot, left ventricular diverticulum, and pulmonary hypoplasia [2]. Other reported associated defects include trisomy 18, cleft lip and palate, neural tube defects, hydrocephaly, pulmonary hypoplasia, genitourinary malformation, and abdominal wall defects ranging from diastasis to omphalocele and evisceration of bowel, liver, and heart [4].

The prognosis of complete ectopia cordis depends on the degree of intracardiac and associated malformations. Untreated it is fatal [2]. Typically, surgical management includes a complete survey for additional congenital defects including intracardiac and urgent covering of the exposed organs and viscera with silastic prosthesis [4]. Alphonso N et al. divide the surgical treatment of thoracic ectopia cordis into four steps: (1) soft tissue coverage of heart; (2) reduction of the heart into the chest cavity; (3) treatment of intracardiac defects; (4) reconstruction of chest wall [2].

## Conclusion

Ectopia cordis is a rare congenital malformation with a complicated prognosis related to associated anomalies and prenatal and postnatal management. Given the increasing availability of ultrasound, this case could have been diagnosed early in pregnancy. Sonographers must remember to be vigilant for rare congenital anomalies. In a limited resource setting with difficulties in transportation and limited skilled facilities to care for complicated surgical cases, an antenatal diagnosis would have allowed the physician and parents to have a frank discussion about the prognosis and their options. An emergency caesarean section may have been avoided if the pregnancy was deemed incompatible with life. The mother could have delivered at the tertiary pediatric hospital if surgical correction was deemed feasible and desired by the parents.
